# The Role of Controlled Motivation in the Self-Esteem of Adolescent Students in Physical Education Classes

**DOI:** 10.3390/ijerph182111602

**Published:** 2021-11-04

**Authors:** Alfonso Valero-Valenzuela, Elisa Huéscar, Juan L. Núñez, Jaime León, Luis Conte, Juan Antonio Moreno-Murcia

**Affiliations:** 1 Department of Physical Activity and Sport, CEI Campus Mare Nostrum, University of Murcia, Santiago de la Ribera, 30720 Murcia, Spain; avalero@um.es (A.V.-V.); conte@um.es (L.C.); 2 Sport Research Center, Department of Health Psychology, Miguel Hernández University of Elche, Avda. de la Universidad, s/n, 03202 Elche, Spain; 3 Department of Psychology, Sociology and Social Work, University of Las Palmas de Gran Canaria, C/Santa Juana de Arco, 1, 35004 Las Palmas de Gran Canaria, Spain; juanluis.nunez@ulpgc.es; 4 Department of Education, University of Las Palmas de Gran Canaria, C/Santa Juana de Arco, 1, 35004 Las Palmas de Gran Canaria, Spain; jaime.leon@ulpgc.es; 5 Sport Research Center, Miguel Hernández University of Elche, Avda. de la Universidad, s/n, 03202 Elche, Spain; j.moreno@umh.es

**Keywords:** school, teaching, behaviours, competence, autonomy, relatedness, psychological needs

## Abstract

The aim of this cross-sectional study was to analyse the relationships between the satisfaction of psychological basic needs, physical education, academic controlling motivation, and self-esteem, and to propose a prediction model in line with the postulates from the hierarchical model found in the self-determination theory. The participants were 618 physical education students from primary and secondary school (317 girls and 301 boys) aged between 10 and 14 years old (*M* = 11.62; *SD* = 0.94). The questionnaires basic psychological needs in exercise measurement scale (BPNES), perceived locus of causality scale (PLOC), the academic motivation scale (EME), and physical self-perception profile (PSPP) were used to measure the studied variables. The results showed that autonomy and relatedness significantly and negatively predicted physical education controlling motivation, which predicted a positive and significant academic controlling motivation. This, in turn, negatively and significantly predicted self-esteem. It is concluded that it is essential to avoid controlling motivation to promote the development of a positive self-perception in students.

## 1. Introduction

Numerous authors have highlighted physical education classes as an ideal context for the development of adaptive behaviours in adolescents [1,2], such as the promotion of positive attitudes and prosocial values of students (respect, participation, autonomy, help others, etc.) [3,4]. Thus, in this context, learning new skills, successfully achieving the proposed tasks, and feeling loved and accepted by peers, could be appropriate factors for the promotion of these values. Among them, working on and increasing self-esteem through the practice of physical activity has become a priority in some intervention programs, due to its positive repercussions on health, especially mental health [5].

Self-esteem is the evaluative feeling that people have of themselves [6]. Gergen [7] defines self-esteem as the degree to which people feel positive about themselves. It affects motivation, life satisfaction, and well-being throughout life [8], and can be used as an indicator of emotional health and psychological benefits gained from regular participation in physical activity [5,9]. In this sense, the theoretical construct of physical self-perceptions [5] indicates that positive experiences lead to positive feelings and improved overall self-esteem. In contrast, a low self-esteem is related to different diseases, such as psychiatric disorders, obesity, eating disorders, etc. [10], and can also be an important predictor of depressive symptoms among young people [11].

To help us understand the emergence and development of certain attitudes, emotions, and behaviours in the context of physical education, the Self-Determination Theory (SDT) [12] has been providing an adequate structure for its study for the last two decades [12,13]. Deci and Ryan [14] postulated that behavioural regulation can be based on autonomous, controlled motivation, or on the absence of motivation. These types of motivation are the reasons why people are persistent with the activities they perform and are found along a continuum of self-determination that qualitatively differs in the cause of action.

According to Ryan and Deci [15], autonomous motivation represents the highest level of self-determination. Autonomous motivation is defined as engaging in activities for the interest on, and the satisfaction derived from the activity itself, in the absence of any external incentives (e.g., rewards, praise) [16]. This type of motivation is represented by intrinsic motivation (the person performs the activity because he/she finds it attractive and fun), as well as identified (the person identifies with the value of the activity and has a high degree of willingness to act) and integrated (the person finds the activity congruent with other values and interests in his/her life) regulations [17]. On its part, controlled motivation is represented by extrinsic motivation (the person acts to obtain rewards or avoid externally imposed punishments), and introjected regulation (the person acts to obtain internal rewards such as feeling good in case of success or to avoid anxiety and guilt in case of failure) [18]. Lastly, the absence of motivation is represented by demotivation (the person feels incompetent and uninterested) [19]. In the educational context, there is abundant evidence that autonomous motivation is related to adaptive outcomes such as persistence in the classroom and academic performance [20].

Within this macro-theory, the mini-theory of basic psychological needs considers that human beings need to satisfy three basic psychological needs that are essential for optimal functioning, integration, personal development, and well-being [12]: autonomy, competence, and relatedness to others. In physical-sport activities, when people interact with their environment, they need to feel competent (feeling of mastery of the task), autonomous (feeling of being the initiator of one’s own actions), and related to others (feeling respected by others and the desire to feel connected with them) [21], and there is a positive relationship between them and physical self-concept [22]. The frustration of one or more of these needs is a trigger for the loss of intrinsic motivation and the approach towards demotivation or extrinsic motivation [23]. The feeling of belonging and being linked to the teacher are key pieces in the improvement of self-esteem [24] and are closely related to improvements at the level of physical condition along with personal and social well-being. Therefore, if the student perceives an activity in a way that it manages to provide opportunities for feeling socially integrated, experiencing task mastery, and satisfying the feeling of autonomy, it will increase the autonomous motivation and well-being of their students [25,26].

Much research has been directed towards the study of autonomous motivation and its positive cognitive, affective, and behavioural consequences in the education environment [15,27]. Thus, studies such as that by Hein and Hagger [28] indicated that motives of a more autonomous nature (i.e., when an activity is regulated on the basis of reasons that stem from the student’s own needs or feelings) led to greater self-esteem in young people in physical activity contexts. Coaches can develop the athletes’ psychological well-being through autonomy-supportive coaching behaviours [29]. Baumeister et al. [30] pointed out that high self-esteem led to better school performance. In contrast, a controlling teaching context has been related with the frustration of the three basic psychological needs (autonomy, competence, and social relationships) [31], and these experiences of frustration predispose individuals to a greater perceived fear of failure, challenge avoidance, and low self-esteem [32,33,34]. In this case, the type of motivation underlying this process is controlling, as the learner feels pressured to act by an external force that he or she did not choose, and which does not coincide with his or her interests or needs, or even by internal imperatives. This process usually involves the need for competence and relationship with others, leaving the need for autonomy far behind. For example, the student may perform the tasks despite not choosing them of his own free will, but he knows that with them he will meet the expectation and succeed (competence) and remain integrated in the group (relationship) [35].

In this sense, although there are numerous works that focus on the positive consequences related to autonomous motivation, there are currently very few studies in education that have examined the ones generated by a controlling motivation [36,37,38,39], its link with the satisfaction of basic needs and its relationship with an emotional consequence such as the student’s self-esteem. Considering the importance of self-esteem in students’ physical health and psychological well-being and, based on self-determination theory, the present study aimed to test the predictive power of basic psychological needs satisfaction on controlling motivation in physical education class, and this in turn on academic controlling motivation, to ultimately predict the perceived self-esteem of secondary school students. The following hypotheses were tested:

**Hypothesis** **1** **(H1).** *The satisfaction of basic psychological needs negatively predicts controlling motivation in physical education.*

**Hypothesis** **2** **(H2).** *Controlling motivation in physical education positively predicts controlling motivation at the academic level.*

**Hypothesis** **3** **(H3).** *Academic controlling motivation negatively predicts students’ perceived self-esteem.*

## 2. Materials and Methods

### 2.1. Ethics Statement

This study was approved by the Research Ethics Committee of Universidad Miguel Hernández de Elche (Elche, Spain) (DPS.JMM.01.14) and meets all ethical and legal standards that are applicable to the research of this survey modality.

### 2.2. Participants

The sample, obtained non-randomly and through convenience sampling, consisted of a total of 618 students (301 boys and 317 girls) aged 10–14 years old (*M* = 11.62, *SD* = 0.94) from 24 public primary and secondary schools in various Spanish municipalities with a middle-class socioeconomic status. In Spain, all primary and secondary schools have a very similar curriculum, and the same number of hours is dedicated to physical education throughout the country.

### 2.3. Procedure

For the collection of information, once the study was approved by the Ethics Committee of the institution of the responsible investigator (DPS.JMM.01.14), we contacted the management team of the educational centres to ask for their collaboration in this study. The students were asked for written authorisation from their parents, as they were under-aged. The administration of the final scales was carried out before the beginning of the physical education lesson and took approximately 20 min to complete. The tool used for data collection was Google forms. The physical education teacher supervised the answers, and during the completion process, all the problems that could arise were solved by the teacher.

### 2.4. Measures

Basic Psychological Needs (BPNES). The version translated into Spanish and adapted to physical education [40] of the Basic Psychological Needs in Exercise Measurement Scale (BPNES; [41]) was used. This instrument contained the following heading “In the classes…” followed by 12 items grouped into three factors (four items per factor) measuring the perception of autonomy (e.g., “the exercises we perform match my interests”), the perception of competence (e.g., “I perform the exercises effectively”), and the perception of relatedness to others (e.g., “I feel I can communicate openly with my peers”). The reliability analysis obtained Cronbach’s alpha values for the three measurements taken of 0.73 for the perception of autonomy, 0.77 for the perception of competence, and 0.81 for the perception of social relationships.

Controlling motivation in physical education. Part of the Spanish validated version [42] of the Perceived Locus of Causality Scale by Goudas, Biddle, and Fox [43] was used. The dimensions, composed of four items each, were introjected regulation (e.g., “Because I want the teacher to think I am a good student”), external regulation (e.g., “Because I will get in trouble if I don’t”), and demotivation (e.g., “But I don’t really know why”) were used. The scale was headed by the statement “I participate in this physical education class…” and was answered through a Likert-type scale from 1 (Strongly disagree) to 7 (Strongly agree). Cronbach’s alpha values of 0.73 for introjected regulation, 0.72 for external regulation, and 0.71 for demotivation were obtained.

Academic controlling motivation. From the Spanish version of the EME [44], named Escala de Motivación Educativa (EME-E, Academic Motivation Scale) by Nuñez, Martín-Albo and Navarro [45] we used the dimensions introjected regulation (e.g., “to prove to myself that I am an intelligent person”), external regulation (e.g., “to be able to get a more prestigious job in the future”), and demotivation (e.g., “I honestly do not know; truly, I have the impression of wasting my time”). Each dimension was composed of four items. It was preceded by the previous statement “Why do you go to school?” These reasons were scored according to a seven-point Likert-type scale ranging from 1 (Does not correspond at all) to 7 (Corresponds completely), with an intermediate score of 4 (Corresponds moderately). Internal consistency was 0.82, 0.78, and 0.81, respectively.

Self-esteem. The self-esteem dimension adapted to the academic context of the Physical Self-Perception Profile questionnaire [46,47] was used. The dimension was composed of 5 items (e.g., “I do not feel confident when it comes to participating in activities”). The responses to the instrument were expressed with a Likert-type scale ranging from 1 (Strongly disagree) to 4 (Strongly agree). The internal consistency was 0.77.

### 2.5. Data Analysis

First, a descriptive statistical analysis (mean, standard deviations, asymmetry, and kurtosis) was performed, assuming the univariate normality of the data when values for asymmetry and kurtosis were within −2/+2 and −7/+7, respectively [48]. The internal consistency of each factor was calculated using the Cronbach’s alpha coefficient, which is acceptable when values are greater than 0.70 [49], and the bivariate correlations of all the variables under study. X^2^ and the ratio X^2^/df [50] were used as absolute measures. To verify the relationship between the variables proposed in the study, the two-step maximum likelihood (ML) approach was used, as it allows testing complex relationships between variables (observed and latent) with multiple ways [51]. On the first step (measurement model) a confirmatory factor analysis (CFA) was performed.

On the second step, the structural equation model (SEM) allowed us to test the hypothesised model including all the variables within the same regression model, taking more than one dependent variable, as well as considering the same variable as both dependent and independent (the three dimensions for basic psychological needs, the three dimensions for controlling motivation in physical education, the three dimensions for academic controlling motivation, and the dimension for self-esteem) [51]. For CFA and SEM, the following absolute and incremental indices were used for analysis: Comparative Fit Index (CFI), Tucker–Lewis Index (TLI), and the Root Mean Square Error of Approximation (RMSEA) with its respective Confidence Interval (CI90%). For the cut-off values, CFI and TLI ≥ 0.90, and RMSEA ≤ 0.80 were considered as acceptable [52]. The Confidence Interval at 95% (CI95%) was considered to measure direct and indirect effect among constructs, accepting significance if the CI did not encompass zero. To test multi-group analysis, the structural SEM model was initially assessed in each group separately (basic psychological needs, controlling motivation in physical education, and academic controlling motivation). The Mardia’s multivariate index was used to check the factors’ multivariate normality, accepting it with values lower than 70 [53]. The present research adopted differences in CFI, TLI, and RMSEA to evaluate structural invariance. The data was analysed using the statistical packages SPSS V. 25 and AMOS V. 23 (SPSS Inc., Chicago, IL, USA).

## 3. Results

### 3.1. Descriptive and Correlation Analysis

Descriptive and correlation values are in Table 1. The results revealed that perception of relatedness was the highest ranked variable from the psychological needs satisfaction, with a mean value of 4.55. The participants’ perception of self-esteem obtained a mean value of 2.77. Academic controlling motivation showed a higher value than physical education controlling motivation (4.70 and 4.21, respectively). The asymmetry and kurtosis values were within −2/+2 and −7/+7, respectively, assuming the univariate normality of the data. Cronbach’s alpha yielded acceptable values for all the variables analysed. The correlation analysis revealed significant and positive correlations between perception of autonomy and competence with the rest of the variables. Physical education and academic controlling motivation showed a positive correlation between them, although this correlation was negative with self-esteem.

### 3.2. Structural Regression Analysis

#### 3.2.1. Measurement Model

A structural equation modelling procedure to test the hypothesised model was conducted using various absolute and relative measures of fit calculated. Firstly, a confirmatory factor analysis (measurement model) was used to confirm and trim the constructs for the groups of these items. The factors’ multivariate normality was accepted with a Mardia’s coefficient lower than 70 (35.29). In addition, the multicollinearity assumption was met, since all the bivariate correlations between variables were below 0.85. The following values were obtained: X^2^ (206, N = 276) = 583.7, *p* < 0.001, X^2^/df = 2.83, CFI = 0.92, IFI = 0.92, TLI = 0.90, RMSEA = 0.06, RMSEA 90% CI = 0.05–0.06, SRMR = 0.07. The standardised regression weights ranged between 0.26 and 0.85, were statistically significant, and yielded satisfactory variance of the error.

#### 3.2.2. Structural Equation Model

**Figure 1:**
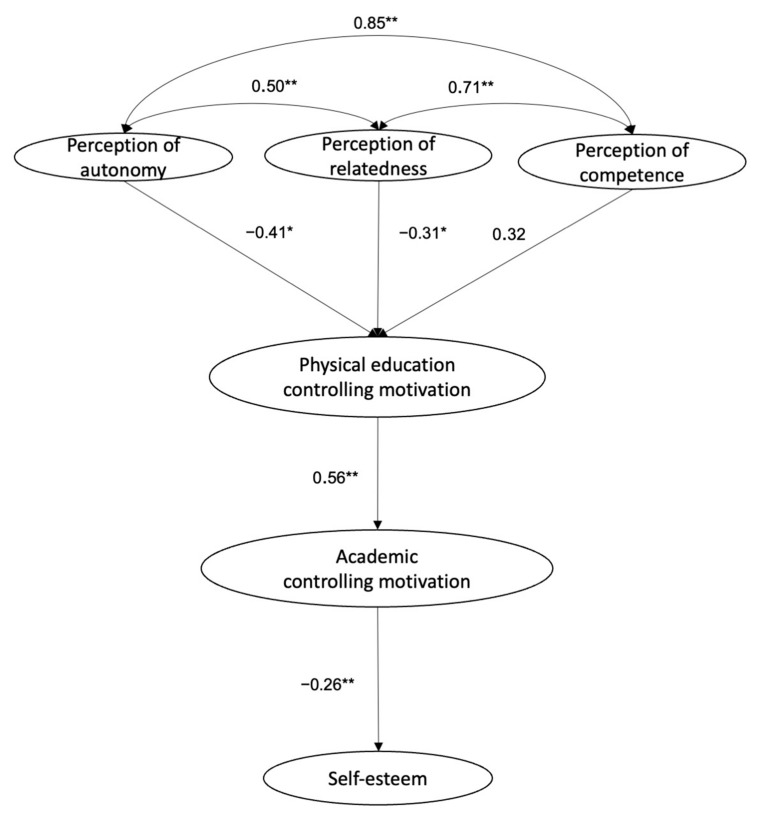
Structural equation model (SEM) that analyses the relationships among perception of autonomy, relatedness, competence, physical education controlling motivation, academic controlling motivation, and self-esteem. All parameters are standardised and significant at *p* < 0.05. Note: * *p* < 0.05, ** *p* < 0.001.

Secondly, a structural equation model was used for testing relationships between indicators and constructs. The model was recursive and identified. The maximum likelihood estimation method was applied in the analysis. The goodness of fit test yielded appropriate fit values according to the established parameters: X^2^ (215, N = 299) = 614.2, *p* < 0.001, X^2^/df = 2.86, CFI = 0.92, IFI = 0.92, TLI = 0.90, RMSEA 0.06, RMSEA 90% CI = 0.05–0.06, SRMR = 0.07. All relationships and standardised regression weights were significant except for controlling motivation in physical education and perception of competence (Figure 1).

Similarly, the contribution of each factor for the prediction of other variables was examined using standardised regression weights. The model’s results (see Figure 1) revealed that perceived autonomy (β = −0.41), relatedness (β = −0.31), and competence (β = 0.32), predicted physical education controlling motivation, which predicted academic controlling motivation (β = 0.56). Finally, this academic controlling motivation predicted self-esteem (β = −0.26), explaining 33%, 31%, and 7% of the variance, respectively.

#### 3.2.3. Indirect Effects

Mediated or indirect effects must be analysed when explaining a model [44]. In the present study, the standardised indirect effects (see Table 2) revealed that only perception of relatedness had a significant indirect effect, in this case negative, with academic controlling motivation (β = −0.17) and a positive one with self-esteem (β = 0.04). Perception of autonomy had a negative effect on academic controlling motivation (β = −0.23) and perception of competence had a positive effect one (β = 0.18). Additionally, perception of autonomy had a positive and indirect effect on self-esteem (β = 0.06), although perception of competence had a negative one (β = −0.05).

## 4. Discussion

Based on the SDT framework [13,54], the aim of the present study was to test, in a sample of adolescent students in physical education classes, the predictive power of the satisfaction of basic psychological needs on controlling motivation, and this in turn, on academic controlling motivation in the academic context, to finally predict adolescent self-esteem.

First, the results of the hypothesised model showed that the three basic psychological needs were positively and significantly related to each other, as postulated by SDT. According to H1, the results showed that perceived controlling motivation in Physical Education classes was negatively predicted by perception of autonomy and relatedness to others, as corroborated previous works [18], thus serving as support for the starting theoretical framework. However, in our work, perception of competence showed a positive relationship with controlling motivation. Therefore, as already pointed out by other works [55,56], the pursuit of competence in physical education classes may be generated by external causal agents (e.g., teacher pressure) rather than internal regulation (e.g., the student performing the tasks because he/she likes learning), giving rise to the less autonomous and more controlling types of motivation. Previous studies in sports, such as Sheenan et al. [57], reported that when tasks were performed based on external reasons such as comparison with others, or outperforming peers, and placing the result as the only incentive for their development, there was a positive relationship with the need for perception of competence, but not with the rest of the variables. This could be possible, as indicated by Smith et al. [58], that of the three basic psychological needs, the one that was least harmed by this type of practice was perception of competence. In this sense, some works have warned of the maladaptive consequences of the use of external reinforcement that feeds the controlling motivation for the promotion of competitiveness in physical education class [18].

The results confirm H2, since a controlling motivation in Physical Education classes predicts controlling motivation in the general academic context. The relationship found is in line with previous research based on Vallerand’s [59] Hierarchical Model regarding the tendency of moderately stable motivational orientations towards each context within the same level of generality [60]. In this case, it is possible that exposure to repeated experiences that create a controlling motivation in physical education classes may have contributed to an extended development of this motivation in the student towards the general academic environment, generating a self-reported controlling motivation with respect to the educational context in which he/she developed. Teachers who use this type of motivation with their students tend to foster in them an external locus of control of perceived causality, offering rewards, threats, and punishments, and unilaterally imposing objectives in advance. On their part, their students’ behaviour is based on obtaining rewards (e.g., passing) or avoiding punishment [18,61]. However, they do not contemplate the importance of learning in the process, enjoying the tasks, or the personal improvement that self-regulation of learning entails [62]. Finally, another risk of using this type of controlling motivation is that the student’s behaviour is always constantly subordinated to the action of the teacher who maintains it, so that when the latter disappears, so does the behaviour. Therefore, the reason could be that the student has not been given the opportunity to build a process of internalisation of learning that he/she can manage by him/herself autonomously without the control of the environmental agents that regulate them [63].

Finally, the results confirm Hypothesis H3, i.e., the predictive and negative power of controlling academic motivation on self-esteem. This result is in line with studies such as Franco et al. [56] and Méndez-Giménez et al. [64], where, although they did not analyse controlling motivation, they reported the existence of a positive relationship between more self-determined motivation and self-esteem. Intervention studies about the development of social and emotional competencies (in line with autonomous motivation using project-based learning) have reported positive changes in self-esteem, apart from better responsible decisions and higher self-awareness in primary education students [65]. A piece of research about service-learning (a pedagogical model focused on achieving curricular goals while providing a community service) showed improvements in the social self-realisation and decisive self-efficacy of Physical Education Teacher Education students, but not in self-esteem [66]. It seems that controlling interventions from teachers could develop a higher controlling motivation and a lower self-esteem in students, and further studies would be very welcome to contrast these statements.

Moreover, thus far there are not many studies that link academic motivation with self-esteem, relating student motives of a more autonomous nature with higher self-esteem [28]. Thus, for example, in the study by Gothe et al. [67] participation in physical activity predicted general self-esteem, or the study by MinHyuk [68] with more than 2000 high school students, where it was found that a better experience in physical education classes was a mediating factor for having higher self-esteem. These results associate the importance of encouraging more autonomous types of motivation to favour a more positive self-esteem [69]. Only one of the basic psychological needs (perception of autonomy) had an indirect effect on the other variables, negative for academic controlling motivation and positive for self-esteem. Previous studies in neuroscience and anger indicated that although all three unsatisfied basic psychological needs were correlated with the trait anger, unsatisfied relatedness was the only factor that was uniquely related to the trait anger [70]. However, in a study about two different models of psychological need satisfaction to well-being in adapted sport athletes, perceived relatedness was the weakest predictor of overall self-esteem, followed by perceived autonomy and competence [71].

The present study shows a series of limitations that should be taken into consideration for future research. In first place, it would be interesting to take into account the social triggers, such as the teacher’s interpersonal style, that may be influencing each of the basic psychological needs and may therefore be generating a more or less controlling motivation in the context of physical education. In addition, the type of sampling used was purposive by accessibility. Future work addressing this issue should be carried out with a more methodologically valid sampling method such as random sampling. Finally, the type of methodological design, cross-sectional and correlational, prevented any type of causal explanation. It would be interesting to carry out longitudinal studies and experimental and/or quasi-experimental designs to test the sequence proposed in this study.

As future lines of research, and within the dynamic process of motivation, it would be interesting to discover whether autonomous motivation has a similar behaviour, as well as to incorporate both the frustration of psychological needs, and the role of the controlling style of the physical education teacher, into the motivational sequence. In addition, it would be important to check the differences according to age in different educational stages, gender, or socio-economic context, as this may be of great importance for future research. As for the practical implications, the teacher should minimise the use of external reinforcements, replacing them by acting as a guide in a process of self-regulation of learning where the process takes precedence over the result, lets the student improve by himself or herself, encourages his or her progress by stressing the value of personal improvement, establishes moments of positive communication and considers mistakes as an opportunity for learning, ultimately helping to promote positive thoughts in the student about himself, developing a higher quality self-esteem. All this, accompanied by the precise tools that the teacher can use to foster the most positive forms of motivation, and decrease motivation of a controlling nature [72], will contribute to the pursuit of greater student well-being in physical education classes.

## 5. Conclusions

In conclusion, the greater importance of self-esteem of the students was explained thanks to higher levels of basic psychological needs (especially relatedness and autonomy perception), and a lower academic and physical education controlling motivation. Moreover, satisfaction of basic psychological needs (especially perception of relatedness) could predict an improvement in the self-esteem of the students. The results of this research suggest the need to promote this type of basic psychological need in physical education and in the educational context the improvement of the student’s self-esteem.

## Figures and Tables

**Table 1:** Mean, standard deviation, and correlations among variables.

Variables	M	SD	a	K	1	2	3	4	5	6
1. Perception of autonomy	3.92	0.75	−0.47	−0.23	-	0.36 **	0.55 **	−0.35 **	−0.31 **	0.16 **
2. Perception of relatedness	4.55	0.60	−1.60	2.35	-	-	0.48 **	0.03	0.06	0.06
3. Perception of competence	4.28	0.65	−0.89	0.28	-	-	-	0.20 **	0.22 **	0.08 *
4. PE controlling motivation	4.21	1.26	−0.15	−0.23	-	-	-	-	0.49 **	−0.34 **
5. Academic controlling motivation	4.70	1.11	−1.22	1.01	-	-	-	-	-	−0.51 **
6. Self-esteem	2.77	0.90	−0.39	−0.86	-	-	-	-	-	-

Note: M = mean; SD = standard deviation; a = asymmetry; K = kurtosis; * *p* < 0.05; ** *p* < 0.01.

**Table 2:** Standardised indirect effects of the variables.

Variables	B
Perception of autonomy → academic controlling motivation	−0.23
Perception of autonomy → self-esteem	0.06
Perception of relatedness → academic controlling motivation	−0.17 *
Perception of relatedness → self-esteem	0.04 *
Perception of competence → academic controlling motivation	0.18
Perception of competence → self-esteem	−0.05

Note: * *p* < 0.05.

## Data Availability

The data sets analyzed during the current study are available from the corresponding author on reasonable request.
